# Podocyte-Specific Deletion of MCP-1 Fails to Protect against Angiotensin II- or Adriamycin-Induced Glomerular Disease

**DOI:** 10.3390/ijms25094987

**Published:** 2024-05-03

**Authors:** Corry D. Bondi, Hannah L. Hartman, Brittney M. Rush, Roderick J. Tan

**Affiliations:** Department of Medicine, University of Pittsburgh, Pittsburgh, PA 152671, USA; hlh51@pitt.edu (H.L.H.); garciabm2@upmc.edu (B.M.R.); tanr@pitt.edu (R.J.T.)

**Keywords:** glomerular injury, chronic kidney disease, proteinuria, podocyte, CCL2, CCR2

## Abstract

Investigating the role of podocytes in proteinuric disease is imperative to address the increasing global burden of chronic kidney disease (CKD). Studies strongly implicate increased levels of monocyte chemoattractant protein-1 (MCP-1/CCL2) in proteinuric CKD. Since podocytes express the receptor for MCP-1 (i.e., CCR2), we hypothesized that podocyte-specific MCP-1 production in response to stimuli could activate its receptor in an autocrine manner, leading to further podocyte injury. To test this hypothesis, we generated podocyte-specific MCP-1 knockout mice (Podo-*Mcp-1^fl/fl^*) and exposed them to proteinuric injury induced by either angiotensin II (Ang II; 1.5 mg/kg/d, osmotic minipump) or Adriamycin (Adr; 18 mg/kg, intravenous bolus). At baseline, there were no between-group differences in body weight, histology, albuminuria, and podocyte markers. After 28 days, there were no between-group differences in survival, change in body weight, albuminuria, kidney function, glomerular injury, and tubulointerstitial fibrosis. The lack of protection in the knockout mice suggests that podocyte-specific MCP-1 production is not a major contributor to either Ang II- or Adr-induced glomerular disease, implicating that another cell type is the source of pathogenic MCP-1 production in CKD.

## 1. Introduction

Chronic kidney disease (CKD) is an increasing global burden affecting nearly a billion people [1,2,3]. It is characterized by a permanent reduction in kidney function, and for many, it can ultimately lead to end-stage renal disease (ESRD) and the need for dialysis and transplantation.

Podocytes are specialized epithelial cells that line the glomerular capillaries. Their interdigitating foot processes form slit diaphragms which act as barriers to prevent proteins, such as albumin, from entering the filtrate [4,5]. Podocyte injury or loss leads to slit-diaphragm disruption, resulting in proteinuria, a known risk factor for CKD progression [6,7]. Therefore, investigating the role of podocytes in the pathophysiology of proteinuric disease is imperative to remedy the increasing global burden of CKD.

Monocyte chemoattractant protein-1 (MCP-1/CCL2), a member of the C-C motif chemokine family, and its cognate receptor, the C-C motif chemokine receptor 2 (CCR2), are produced and expressed by podocytes and both may be involved in proteinuric CKD [8,9,10,11,12,13,14,15,16,17,18,19,20,21,22,23,24]. The CCR2 protein and transcripts are increased in biopsy samples taken from nephrotic patients [15,25]. CCR2 knockout and chemical inhibition are effective in reducing Adriamycin (Adr)-induced glomerular injury and proteinuria in mice [25,26]. In diabetic mice, CCR2 knockout and chemical inhibition protect against proteinuria [16]. While global CCR2 knockout is protective, targeted podocyte-specific CCR2 receptor re-expression sensitized mice to glomerular injury and proteinuria [17]. 

In addition to increased *CCR2* levels, *MCP-1* transcripts are also upregulated, particularly in focal segmental glomerulosclerosis [25]. Clinically, MCP-1 levels correlate with the extent of proteinuria and with a decline in renal function [18,19,20,21,22,27,28,29,30]. Ang II- and Adr-induced injuries increase MCP-1 expression or its mRNA in vivo. Adr exposure decreases glomerular *Nphs1* (nephrin) mRNA expression over the course of 28 days while *Mcp-1* transcripts steadily increase [25]. Angiotensin II (Ang II) infusion increases glomerular MCP-1 expression in rats [31]. In diabetic mice, urinary levels of MCP-1 correlate with severity of albuminuria and knocking out MCP-1 protects against proteinuria [12,15,32]. Other studies show that podocytes are adversely affected by MCP-1. For example, podocytes exposed to MCP-1 downregulate nephrin, leading to barrier disruption and proteinuria as well as inducing apoptosis [10,15,24]. Inhibition of MCP-1 blocks podocyte injury and proteinuria in podocyte-specific Twist1 knockout mice exposed to glomerular injury [33]. Collectively, these studies strongly implicate the MCP-1/CCR2 system in proteinuric CKD, but these studies fail to examine the primary source of MCP-1 involved in the development of glomerular injury.

The objective of our study was to determine the source of MCP-1 that contributes to glomerular disease. Autocrine activity is possible since podocytes can express both MCP-1 and its receptor, CCR2. We hypothesized that podocyte-specific MCP-1 production leads to progressive glomerular injury and proteinuria. To fully discriminate the effects elicited by the podocyte generation of MCP-1, we generated podocyte-specific conditional MCP-1 knockout (Podo-*Mcp-1^fl/fl^*) mice. We subjected these mice and their wild-type littermates to either angiotensin II (Ang II) or Adriamycin (Adr) to induce glomerular injury. We assessed survival, change in body weight, albuminuria, kidney function, glomerular injury, and tubulointerstitial fibrosis, since both models produce glomerular injury followed by proteinuria and fibrosis within 28 days [25,34]. Overall, our results show that podocyte-derived MCP-1 is dispensable for the development of glomerular injury in our models.

## 2. Results

### 2.1. Generation of Podocyte-Specific MCP-1 (Podo-Mcp-1^fl/fl^) Knockout Mice

**Figure 1:**
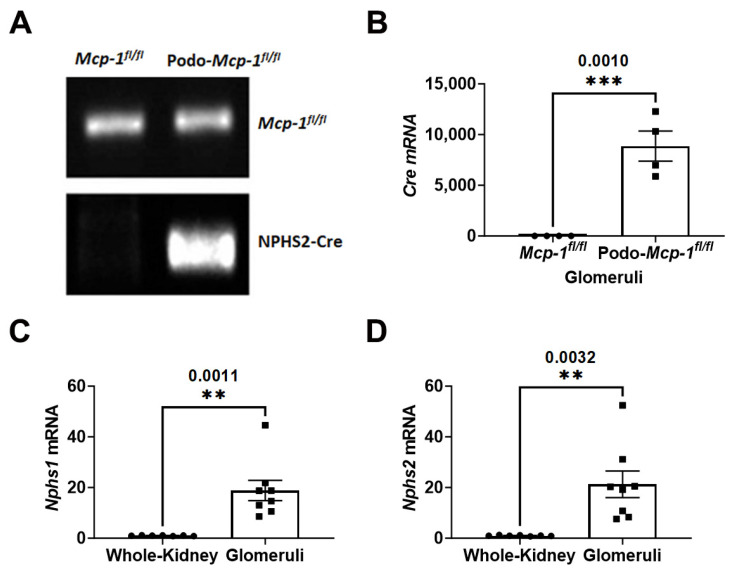
Generation of podocyte-specific MCP-1 knockout mice. (**A**) Podo-*Mcp-1^fl/fl^* mice harbor both the *Mcp-1* floxed allele and Cre recombinase under control of the *NPHS2* (podocin) promoter. Control littermates (*Mcp-1^fl/fl^*) lack the *NPHS2*-Cre recombinase transgene. (**B**) Significant *Cre* mRNA expression in isolated glomeruli from Podo-*Mcp-1^fl/fl^* compared to *Mcp-1^fl/fl^* mice. mRNA expression of podocyte-specific markers, (**C**) *Nphs1* (nephrin) and (**D**) *Nphs2* (podocin), in the glomeruli compared to whole-kidney homogenates showing significant enrichment in glomerular preparations. (**B**–**D**) Unpaired Student’s *t*-test (two-tailed). Individual data point represents a mouse. Mean ± S.E.M. Actual *p* values are presented on graphs. Threshold for significance was *p* < 0.05 with ** *p* < 0.01 and *** *p* < 0.001.

Podocyte-specific MCP-1 knockout mice (Podo-*Mcp-1^fl/fl^*) were generated by crossing mice expressing the Cre recombinase under control of the podocin (*NPHS2*) promoter to *Mcp-1^fl/fl^* mice. Genotype analysis confirmed that these mice harbor both the *Mcp-1* floxed allele and podocin-Cre recombinase, unlike their control littermates (*Mcp-1^fl/fl^*) (Figure 1A). To characterize the knockout model, glomeruli were isolated from whole kidneys of Podo-*Mcp-1^fl/fl^* and *Mcp-1^fl/fl^* mice. The glomerular expression of *Cre* mRNA was significantly, about 9000-fold (8864 ± 1479 compared to 1.000 ± 0.07707, *p* = 0.0010), higher in these mice compared to control littermates (Figure 1B). The quality of the glomerular preparation was confirmed by a significantly higher expression of *Nphs1* (nephrin) (18.85 ± 4.003 compared to 1.000 ± 0.05381, *p* = 0.0011) and *Nphs2* (podocin) (21.27 ± 5.238 compared to 1.000 ± 0.06575, *p* = 0.0032) compared to whole-kidney homogenates (Figure 1C,D).

### 2.2. Kidneys from Podo-Mcp-1^fl/fl^ Mice Exhibit no Abnormalities at Baseline

**Figure 2:**
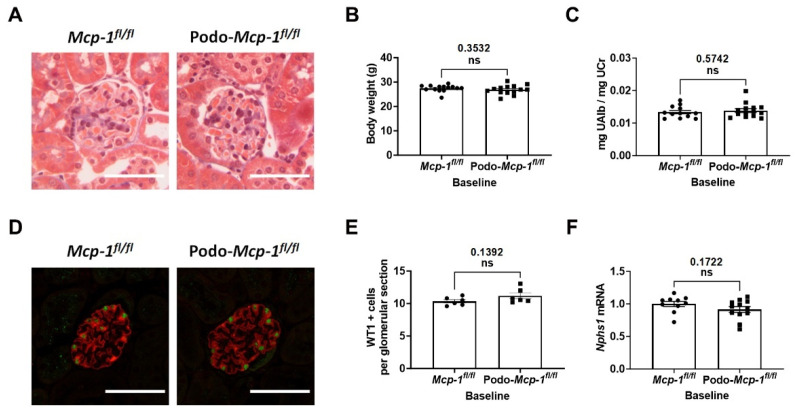
Kidneys from Podo-*Mcp-1^fl/fl^* mice exhibit no abnormalities at baseline. (**A**) Representative Masson’s trichrome stain images are shown. Histological analysis revealed no observable glomerular or tubular abnormalities in control and knockout mice. Scale bar = 50 µm. There were no significant differences in (**B**) body weight and (**C**) albuminuria between groups. (**D**) Representative glomeruli immunofluorescence images displaying WT1 (Green) and nephrin (Red). Scale bar = 50 µm. No between-group differences in the (**E**) number of WT1 positive cells and mRNA expression of (**F**) *Nphs1* (nephrin). (**B**,**C**,**E**,**F**) Unpaired Student’s *t*-test (two-tailed). Individual data point represents a mouse. Mean ± S.E.M. Actual *p* values are presented on graphs. Threshold for significance was *p* < 0.05 with ns = statistically non-significant, *p* > 0.05.

We first examined whether knocking out MCP-1 in the podocytes resulted in a baseline phenotype. Histological analysis with Masson’s trichrome stain revealed no observable glomerular or tubular abnormalities (Figure 2A). There were no significant differences in body weight (26.84 ± 0.5015 g compared to 27.42 ± 0.3639 g, *p* = 0.3532) and albuminuria (0.01384 ± 0.0005903 mg UAlb/mg UCr compared to 0.01338 ± 0.0005368 mg UAlb/mg UCr, *p* = 0.5742) between the groups (Figure 2B,C). Representative immunofluorescence images of glomeruli show intact nephrin network and WT1 positive cells (Figure 2D). The number of WT1-positive cells (11.18 ± 0.4562 compared to 10.33 ± 0.2574, *p* = 0.1392) and mRNA expression of *Nphs1* (nephrin) (0.9147 ± 0.0493 compared to 1.000 ± 0.03966, *p* = 0.1722) were also similar between groups (Figure 2E,F). These results suggest that knocking out MCP-1 in the podocytes did not result in any observable developmental abnormalities nor any apparent injury to the kidney or glomeruli.

### 2.3. Knocking out MCP-1 from the Podocyte Did Not Affect Survival, Change in Body Weight, Albuminuria, and Kidney Function in Two Models of Proteinuric CKD

**Figure 3:**
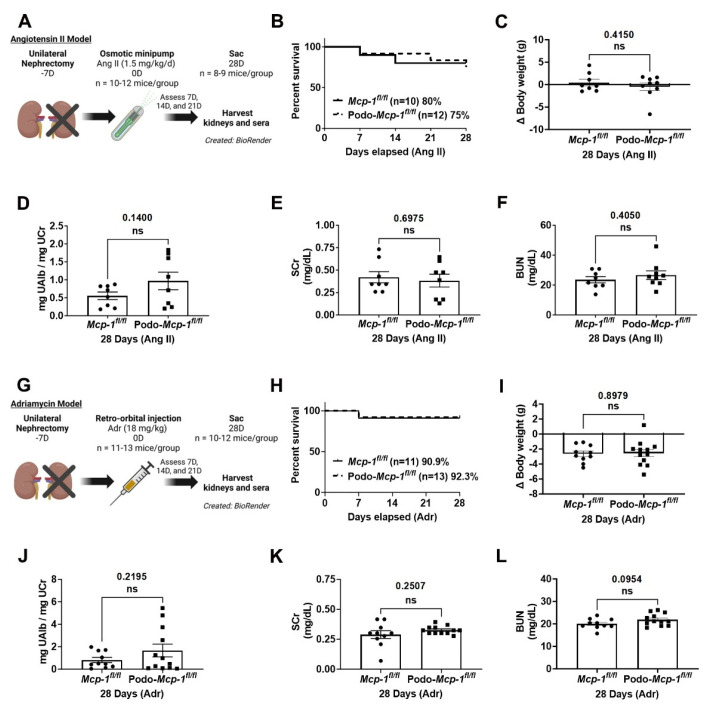
Control and knockout mice exhibit similar survival, change in body weight, albuminuria, and kidney function after Ang II or Adr exposure. To evaluate whether podocyte-specific MCP-1 contributes to podocyte injury, (**A**) Ang II (1.5 mg/kg/day, osmotic minipump) or (**G**) Adr (18 mg/kg, intravenous bolus) were given to induce glomerular injury. Results are reported at 28 days. Experimental schemes created with BioRender. (**B**,**H**) Kaplan–Meier curves show survival proportions are similar between groups. No between-group differences were detected in (**C**,**I**) change in body weight (from Day 0 to Day 28), (**D**,**J**) albuminuria, (**E**,**K**) serum creatinine (SCr), and (**F**,**L**) blood urea nitrogen (BUN). For change in body weight in Ang II-exposed mice, the weight of the empty osmotic pump was subtracted from Day 28 body weight to account for the added weight attributed to by the pump. (**B**,**H**) Log-rank test and (**C**–**F**,**I**–**L**) Unpaired Student’s *t*-test (two-tailed). Individual data point represents a mouse. Mean ± S.E.M. Actual *p* values are presented on graphs. Threshold for significance was *p* < 0.05 with ns = statistically non-significant, *p* > 0.05.

To evaluate whether podocyte-specific MCP-1 production was necessary to the development of glomerular injury, specifically focal segmental glomerulosclerosis, mice were exposed to Ang II (1.5 mg/kg/day, osmotic minipump) or Adr (18 mg/kg, intravenous bolus) to induce podocyte injury (Figure 3A,G). A unilateral nephrectomy was performed 7 days prior to these exposures to sensitize the mice, which were on the injury resistant C57BL/6J background, to kidney damage [35]. The exposure (i.e., Day 0) began with 10 control and 12 knockout mice in the Ang II experiment and the Adr experiment had 11 control and 13 knockout mice. A total of eight control and nine knockout mice in the Ang II model survived to Day 28 and ten control mice and twelve knockout mice in the Adr model reached the study endpoint. Survival analysis demonstrated the two groups had similar survival proportions (Ang II: 75% compared to 80%, *p* = 0.8382 and Adr: 92.3% compared to 90.9%, *p* = 0.9038) (Figure 3B,H). At 28 days, there were no differences in change in body weight (i.e., from Day 0 to Day 28) (Ang II: −0.4778 ± 0.8534 g compared to 0.4750 ± 0.7275 g, *p* = 0.4150 and Adr: −2.558 ± 0.4869 g compared to −2.640 ± 0.3615 g, *p* = 0.8979) or albuminuria (Ang II: 0.9698 ± 0.2436 mg UAlb/mg UCr compared to 0.5554 ± 0.1040 mg UAlb/mg UCr, *p* = 0.1400 and Adr: 1.658 ± 0.5713 mg UAlb/mg UCr compared to 0.8195 ± 0.2279 mg UAlb/mg UCr, *p* = 0.2195) between control and knockout mice in either model (Figure 3C,D,I,J). Similarly, there were no between-group differences in serum creatinine (SCr) (Ang II: 0.3825 ± 0.0717 mg/dL compared to 0.4203 ± 0.0625 mg/dL, *p* = 0.6975 and Adr: 0.3260 ± 0.0096 mg/dL compared to 0.2894 ± 0.0321 mg/dL, *p* = 0.2507) and blood urea nitrogen (BUN) (Ang II: 26.66 ± 2.821 mg/dL compared to 23.57 ± 2.122 mg/dL, *p* = 0.4050 and Adr: 21.92 ± 0.7678 mg/dL compared to 20.05 ± 0.7177 mg/dL, *p* = 0.0954) (Figure 3E,F,K,L). These results suggest that podocyte-specific deletion of MCP-1 did not protect against Ang II- or Adr-induced injury.

### 2.4. Knocking out MCP-1 Did Not Protect against Glomerular Injury

**Figure 4:**
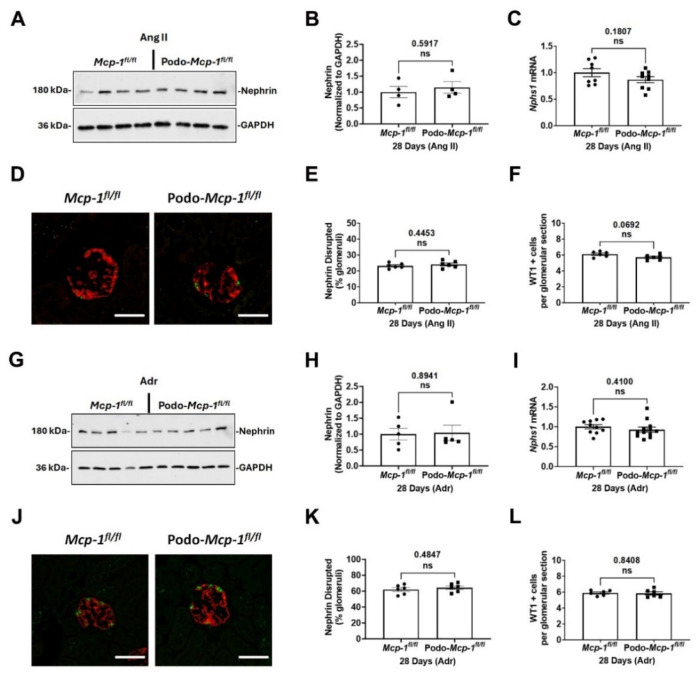
Nephrin expression and podocyte number are similar in control and knockout mice exposed to Ang II or Adr. Representative immunoblot images of nephrin protein expression from (**A**) Ang II- or (**G**) Adr-exposed kidneys. (**B**,**H**) Graphs present densitometric analysis of nephrin normalized to GAPDH (loading control). There are no significant between-group differences. (**C**,**I**) *Nphs1* (nephrin) mRNA expression is similar between the groups. (**D**,**J**) Representative glomeruli images displaying WT1 (Green) and nephrin (Red) immunofluorescence staining. Scale bar = 25 µm. (**E**,**K**) The percentage of glomeruli with nephrin disrupted are similar. (**F**,**L**) No significant between-group differences in the number of WT1-positive cells. (**B**,**C**,**E**,**F**,**H**,**I**,**K**,**L**) Unpaired Student’s *t*-test (two-tailed). Individual data point represents a mouse. Mean ± S.E.M. Actual *p* values are presented on graphs. Threshold for significance was *p* < 0.05 with ns = statistically non-significant, *p* > 0.05.

By differing mechanisms, Ang II and Adr exposures result in podocyte injury and loss leading to disruption of the slit diaphragm and subsequent proteinuria [35,36,37,38]. Nephrin is an integral component of the slit diaphragm [39,40]. Immunoblot analysis of nephrin protein expression revealed no significant differences (Ang II: 1.145 ± 0.1837 compared to 1.000 ± 0.1786, *p* = 0.5917 and Adr: 1.042 ± 0.2431 compared to 1.000 ± 0.1830, *p* = 0.8941) (Figure 4A,B,G,H). Also, there were no between-group differences in *Nphs1* mRNA expression (Ang II: 0.8675 ± 0.05662 compared to 1.000 ± 0.07730, *p* = 0.1807 and Adr: 0.9294 ± 0.06316 compared to 1.000 ± 0.05190, *p* = 0.4100) (Figure 4C,I). Furthermore, the extent of glomerular injury as determined by assessing nephrin disruption was also similar (Ang II: 24.10 ± 0.8772% compared to 23.23 ± 0.6589%, *p* = 0.4453 and Adr: 64.38 ± 2.176% compared to 62.03 ± 2.392%, *p* = 0.4847) (Figure 4E,K). Since glomerular injury results in podocyte loss, WT1 counts were performed to assess the number of remaining podocytes in the injured glomeruli. No between-group differences were detected (Ang II: 5.708 ± 0.1393 compared to 6.108 ± 0.1387, *p* = 0.0692 and Adr: 5.850 ± 0.2029 compared to 5.900 ± 0.1329, *p* = 0.8408) (Figure 4F,L). These results suggest knocking out MCP-1 from the podocyte did not protect against podocyte injury.

### 2.5. Knocking Out MCP-1 Did Not Protect against Tubulointerstitial Fibrosis

**Figure 5:**
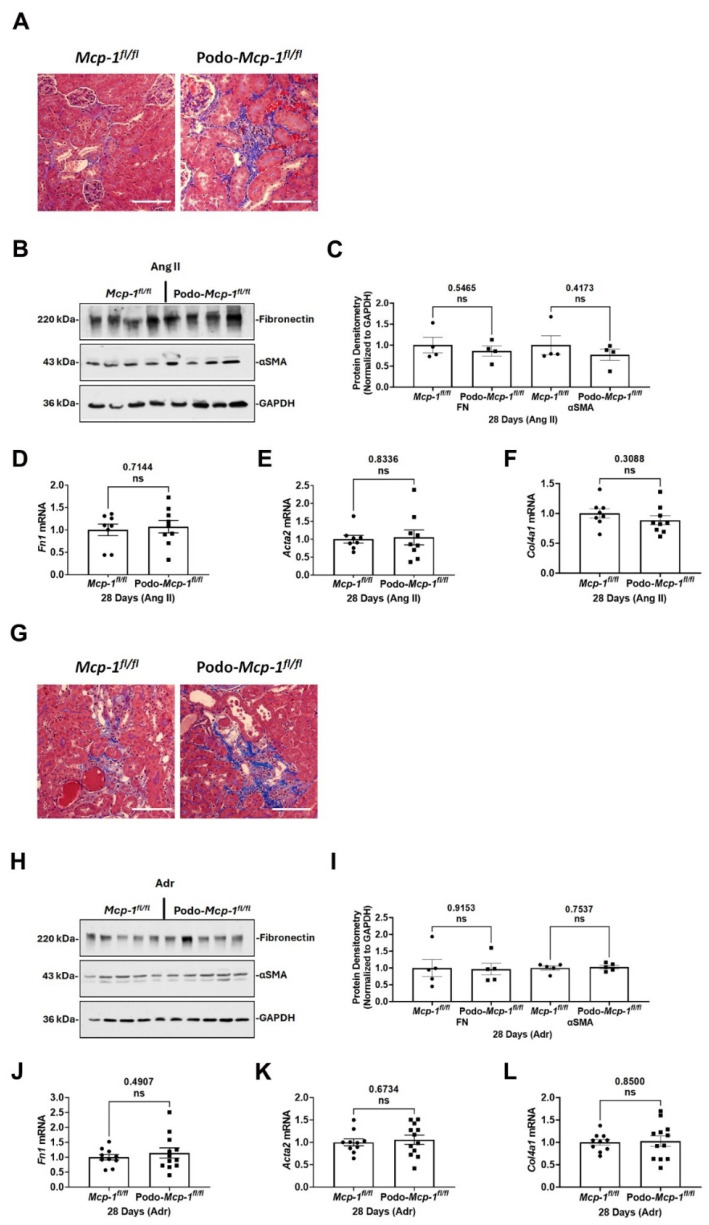
Renal fibrosis was similar in control and knockout mice exposed to Ang II or Adr. (**A**,**G**) Representative Masson’s trichrome stain images. Histological analysis revealed observable interstitial fibrosis. Scale bar = 100 µm. (**B**,**H**) Representative immunoblot images of fibronectin and alpha smooth muscle actin (αSMA) protein expression. (**C**,**I**) Graphs present densitometric analysis of both fibronectin and αSMA normalized to GAPDH (loading control). (**D**–**F**,**J**–**L**) qPCR analysis of *Fn1* (fibronectin), *Acta2* (actin alpha 2, smooth muscle), and *Col4a1* (collagen, type IV, alpha 1). There were no significant between-group differences in *Fn1*, *Acta2*, and *Col4a1* expression for either exposure. (**C**–**F**,**I**–**L**) Unpaired Student’s *t*-test (two-tailed). Individual data point represents a mouse. Mean ± S.E.M. Actual *p* values are presented on graphs. Threshold for significance was *p* < 0.05 with ns = statistically non-significant, *p* > 0.05.

In assessing the extent of Ang II- or Adr-induced fibrosis, histological analysis with Masson’s trichrome stain revealed interstitial fibrosis (Figure 5A,G). To quantitate the extent of fibrosis, fibronectin, alpha smooth muscle actin (αSMA), and *Col4a1* (collagen, type IV, alpha 1) were assessed. No between-group differences were detected in fibronectin protein (Ang II: 0.8591 ± 0.1214 compared to 1.000 ± 0.1841, *p* = 0.5465 and Adr: 0.9663 ± 0.1740 compared to 1.000 ± 0.2532, *p* = 0.9153) and *Fn1* mRNA (Ang II: 1.072 ± 0.1393 compared to 1.000 ± 0.1293, *p* = 0.7144 and Adr: 1.144 ± 0.1705 compared to 1.000 ± 0.09224, *p* = 0.4907) for Ang II or Adr exposures (Figure 5B,C,D,H,I,J). Likewise, no differences were detected in αSMA protein (Ang II: 0.7708 ± 0.1345 compared to 1.000 ± 0.2262, *p* = 0.4173 and Adr: 1.026 ± 0.051898 compared to 1.000 ± 0.06069, *p* = 0.7537) and its gene expression (Ang II: 1.052 ± 0.2092 compared to 1.000 ± 0.1070, *p* = 0.8336 and Adr: 1.057 ± 0.1030 compared to 1.000 ± 0.07806, *p* = 0.6734) (Figure 5B,C,E,H,I,K). Furthermore, there were no between-group differences in *Col4a1* mRNA expression (Ang II: 0.8854 ± 0.07647 compared to 1.000 ± 0.07685, *p* = 0.3088 and Adr: 1.027 ± 0.1166 compared to 1.000 ± 0.06313, *p* = 0.8500) following either exposure (Figure 5F,L). The collective results suggest that knocking out MCP-1 did not protect against Ang II- or Adr-induced tubulointerstitial fibrosis.

### 2.6. Knocking out MCP-1 Did Not Affect mRNA Levels of Mcp-3 and -5

**Figure 6:**
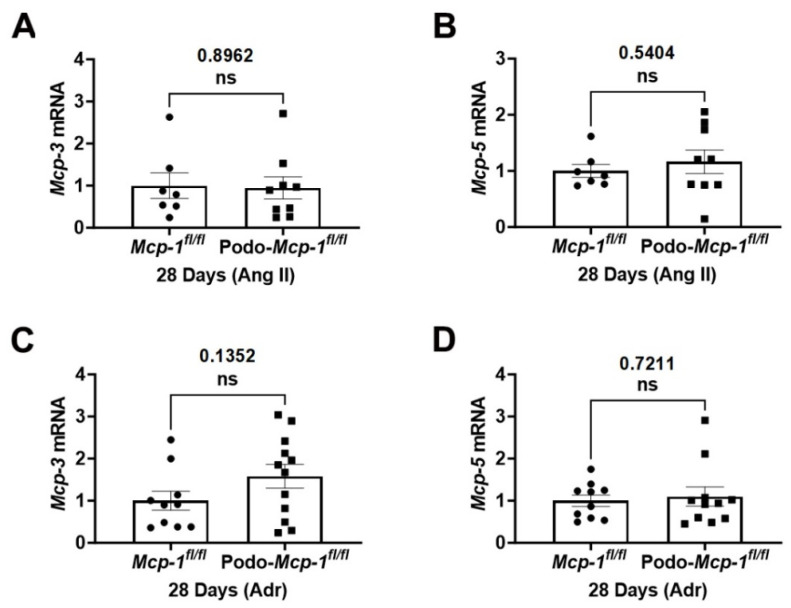
There were no differences in levels of *Mcp-3* and *-5* in control and knockout mice exposed to either proteinuric model. (**A**–**D**) qPCR analysis of *Mcp-3* (monocyte chemoattractant rotein-3) and *Mcp-5* (monocyte chemoattractant protein-5). There were no significant between-group differences for either exposure. (**A**–**D**) Unpaired Student’s *t*-test (two-tailed). Individual data point represents a mouse. Mean ± S.E.M. Actual *p* values are presented on graphs. Threshold for significance was *p* < 0.05 with ns = statistically non-significant, *p* > 0.05.

Podocyte-specific MCP-1 knockout enabled us to specifically interrogate the effect elicited by MCP-1. However, other chemokines such as MCP-3 and -5 interact with CCR2 and compensate for a lack of MCP-1 [41]. Therefore, we assessed the mRNA levels of *Mcp-3* and *Mcp-5*. No significant between-group differences in *Mcp-3* (Ang II: 0.9468 ± 0.2612 compared to 1.000 ± 0.3053, *p* = 0.8962 and Adr: 1.579 ± 0.2819 compared to 1.000 ± 0.2258, *p* = 0.1352) and *Mcp-5* (Ang II: 1.163 ± 0.2093 compared to 1.000 ± 0.1170, *p* = 0.5404 and Adr: 1.098 ± 0.2275 compared to 1.000 ± 0.1347, *p* = 0.7211) were detected for either exposure (Figure 6A–D). These results suggest that neither *Mcp-3* nor *Mcp-5* are upregulated in knockout mice to compensate for a lack of podocyte-derived MCP-1 during disease.

## 3. Discussion

In this study, we evaluated whether podocyte-specific MCP-1 production contributes to proteinuric CKD. To do so, we utilized two well-defined models of proteinuric CKD in podocyte specific MCP-1 knockout mice (Figure 1). At baseline, based on our assessments, there was no apparent pathological phenotype (Figure 2). Following exposure, we evaluated outcomes such as survival, change in body weight, albuminuria, kidney function, glomerular injury, and tubulointerstitial fibrosis using a variety of methods (Figure 3, Figure 4 and Figure 5). Overall, our results suggested that knocking out MCP-1 from the podocyte did not afford protection against Ang II- and Adr-induced injury, suggesting that podocyte-specific expression of MCP-1 is not a major contributor to disease.

The MCP-1/CCR2 system is involved in proteinuric CKD. Podocytes produce MCP-1 in response to stimuli and express its cognate receptor, CCR2, suggesting potential autocrine actions [8,9,15,24]. In addition to its traditional role in the inflammatory response, MCP-1 has other effects on podocytes, such as inducing apoptosis and nephrin loss and increasing their migratory capacity [9,10,24]. Since other chemokines, such as MCP-3 and -5, also interact with CCR2, strategies that block or knockout CCR2 do not fully discriminate between these chemokines, thereby failing to delineate the effects of MCP-1. Therefore, we chose to knockout MCP-1 from the podocyte, thus allowing us to specifically interrogate the effect elicited by MCP-1. Also, this approach allowed us to capture its non-receptor-related actions, since MCP-1 was shown to activate NF-κB in a CCR2-independent manner in vitro in tubular epithelial cells [42]. It was possible that other chemokines such as MCP-3 may compensate for a lack of MCP-1, thus hiding its role in certain diseases [41]. To investigate this, we assessed *Mcp-3* and *Mcp-5* expression in mice exposed to both proteinuric CKD models and found that the expression levels were similar between the groups, suggesting that there is not a compensatory increase in their expression in response to *Mcp-1* knockout (Figure 6).

We chose two well-documented models of proteinuric CKD to test our hypothesis. Chronic angiotensin II infusion is relevant because the renin–angiotensin system is implicated in inducing renal damage in many types of CKD [37,38]. Treatment of diabetic nephropathy patients with angiotensin-converting enzyme inhibitors improves kidney function and reduces urinary MCP-1 levels [43]. Research shows that podocytes express the Ang II receptor type 1 and overexpression results in proteinuria and glomerulosclerosis in transgenic rats [44,45]. Nephrin expression and distribution are sensitive to Ang II levels and increased Ang II levels induce podocyte apoptosis and subsequent proteinuria in Sprague–Dawley rats [46]. Ang II infusion increases glomerular MCP-1 expression in rats [31]. In cultured mouse podocytes, high glucose increases *Mcp-1* mRNA expression, and treatment with an Ang II receptor inhibitor inhibits increases in *Mcp-1* mRNA expression [47]. In our study, after 28 days of continuous infusion, we found no between-group differences in survival, change in body weight, albuminuria, kidney function, glomerular injury, and tubulointerstitial fibrosis. This suggests podocyte-specific MCP-1 production is not a major contributor to Ang II-induced injury (Figure 3, Figure 4 and Figure 5).

Adriamycin exposure causes proteinuric CKD and is used as a model of focal segmental glomerulosclerosis [35,36]. The toxic effects of Adr result in podocyte foot process effacement and disruption of the glomerular filtration barrier leading to proteinuric CKD [35,48]. Adr exposure induces MCP-1 secretion in isolated glomeruli from BALB/c mice in vitro as well as steadily increases *Mcp-1* mRNA expression over 28 days in vivo [25]. It is known that C57BL/6 mice are more resistant to glomerular injury by Adr than BALB/c mice [35]. To overcome this resistance, we used 18 mg/kg in our study coupled with unilateral nephrectomy, as previously described [34,35]. Adr also induces the release of MCP-1 from tubular cells [35], which could likely have occurred in our experimental mice. However, Podo-*Mcp-1^fl/fl^* mice exhibited no difference in Adr injury compared to control littermates, again supporting our assertion that podocyte-specific MCP-1 production is not a major contributor to glomerular injury.

In diabetic mice, knocking out MCP-1 prevents nephrin loss and protected against proteinuria [12,15]. Cultured podocytes exposed to MCP-1 downregulate nephrin in a CCR2-Rho-kinase dependent mechanism [15]. These findings suggest that MCP-1 contributes to both nephrin loss and glomerular permeability via CCR2. In both of our exposure models, podocyte-specific MCP-1 knockout mice had similar nephrin protein and mRNA expression compared to control littermates. Additionally, the number of remaining podocytes was not different between the groups (Figure 4)

The MCP-1/CCR2 system is involved in kidney fibrosis [49]. The profibrotic sclerotic effects of MCP-1 were examined in MCP-1 deficient or intact streptozotocin-induced diabetic mice. Knocking out MCP-1 decreases fibronectin protein expression [50]. However, in our podocyte-specific knockout mice, we show similar fibrotic deposition, fibronectin and αSMA protein levels, and gene expression between the groups suggesting a lack of protection against fibrosis in the knockout mice (Figure 5).

If kidney MCP-1 promotes disease in CKD, it is likely being generated by other cell types besides the podocyte. Other potential sources of MCP-1 include mesangial, tubular epithelial, and endothelial cells. Indeed, mesangial cells produce MCP-1 via an NF-κB dependent pathway in response to high glucose and mechanical stretch [51]. Several studies have shown that MCP-1 is upregulated in tubules during DN and other proteinuric diseases [19,20,21,23,52]. Lastly, MCP-1 is also produced by injured glomerular endothelial cells [53]. These cells could cause podocyte injury through a paracrine pathway and will be evaluated in future studies.

Our study has several strengths. First, the use of two clinically relevant models of glomerular injury with different mechanisms of injury increases the generalizability of our findings. Second, our use of transgenic mice is the only way to definitively test whether podocytes are the key producers of pathologic MCP-1 in vivo. Pharmacologic or chemical inhibitors are not specific for determining the source of MCP-1 and may have off-target effects. Third, we examined several indices of biochemical injury (albuminuria, serum creatinine, and BUN), cellular injury (WT1 and nephrin), and fibrosis (fibronectin, αSMA, and histology), with none of the results showing statistical significance.

Our study also has several limitations. First, because MCP-1 knockout was induced via constitutive activity of the podocin promoter, we cannot definitively rule out a developmental defect. However, we did not observe any baseline defects in renal function or histology (Figure 2), and the global MCP-1 knockout mouse has no observed renal phenotype [12,15]. Second, while our models are accepted methods of studying glomerular injury, we did not include a model of diabetic nephropathy. Third, our study only included male mice since female mice are resistant to our chosen disease models.

Overall, this study used clinically relevant models of glomerular injury in conditional knockout mice to elucidate the role of podocyte-derived MCP-1 production in proteinuric CKD. Our findings indicate that podocyte-derived MCP-1 is not necessary for disease development in our model systems. Other sources of MCP-1 are likely to play a role in disease pathogenesis. These results provide a valuable contribution to furthering the understanding of podocyte-specific MCP-1 in CKD pathogenesis.

## 4. Materials and Methods

### 4.1. Animals

IACUC protocol approval was obtained at the University of Pittsburgh. Control littermates and Podo-*Mcp-1^fl/fl^* mice were afforded the ethical and scientific standards recommended by the Guide for the Care and Use of Laboratory Animals of the National Institutes of Health. ARRIVE guidelines were followed [54]. Male 8- to 12-week-old mice were housed (maximum 4 males/cage) in plastic cages with wood chips in the animal facility at 72 °C and 39.6% humidity on a 12:12 h light–dark cycle with water and Prolab^®^ IsoPro^®^ RMH 3000 5P75 diet (PMI Nutrition International, LLC, Arden Hills, MN, USA) provided ad libitum.

To generate Cre-lox conditional knockout (Podo-*Mcp-1^fl/fl^*) mice, female B6.Cg-*Ccl2^tm1.1Pame^*/J containing the *lox*P sites flanking exons 2 and 3 of the C-C motif ligand 2 (*Ccl2/Mcp-1*) (#016849, Jackson Laboratory, Bar Harbor, ME) [55] were crossed with male B6.Cg-Tg(NPHS2-Cre)295Lbh/J expressing Cre recombinase under the control of the podocin (*NPHS2*) promoter (#008205, Jackson Laboratory) [56]. All mice are on a C57BL/6J background.

### 4.2. Antibodies

Primary antibodies utilized for immunoblot (IB) and immunofluorescence (IF): alpha smooth muscle actin (αSMA) (IB: 1:1000, #14395-1-AP, Proteintech), fibronectin (IB: 1:1000, #F3648, MilliporeSigma, Burlington, MA, USA), GAPDH HRP conjugated (IB: 1:10,000, #HRP-60004, Proteintech, Rosemont, IL, USA), nephrin (IB: 1:1000, IF: 1:200, #20R-NP002, Fitzgerald Industries International, Acton, MA, USA), and WT1 (IF: 1:100, #PA5-16879, Invitrogen, Rockford, IL, USA).

### 4.3. Proteinuria Model

Mice were anesthetized with an intraperitoneal injection of ketamine (100 mg/kg) and xylazine (10 mg/kg) before being subjected to abdominal incision and unilateral nephrectomy of the left kidney 7 days prior to the start of a proteinuria model. The exposure (i.e., Day 0) began with 10 control and 12 knockout mice in the Ang II experiment and the Adr experiment had 11 control and 13 knockout mice. For the angiotensin II model, osmotic minipumps (Model 2004, Alzet, Cupertino, CA, USA) containing angiotensin II (Ang II) (1.5 mg/kg/day, #4006473, Bachem, Torrance, CA, USA) in 0.01 M acetic acid were subcutaneously implanted into the mice as previously described (Figure 3A) [34]. To determine the change in body weight for the Ang II groups, the weight of the empty osmotic pump was subtracted from Day 28 body weight to account for the added weight attributed to the pump. For the Adriamycin model, Adriamycin (Adr) (18 mg/kg, doxorubicin-HCl, #D1515, MilliporeSigma) was delivered via a single intravenous, retro-orbital injection (Figure 3G) [57]. A total of 8 control and 9 knockout mice in the Ang II model survived to Day 28 and 10 control mice and 12 knockout mice in the Adr model reached the study endpoint. The mice were euthanized with a dose of ketamine (300 mg/kg) and xylazine (30 mg/kg) followed by cervical dislocation at 28 days post implantation or injection, at which time kidneys and sera were harvested.

### 4.4. Biochemical Measurements

Spot urine collection was performed, and urine albumin excretion was determined with a mouse albumin ELISA kit (Bethyl Laboratories, Worthington, TX, USA). Urine and serum creatinine levels were assayed with the Creatinine (Enzymatic) Reagent Set (#C7548-120, Pointe Scientific, Canton, MI, USA). Blood urea nitrogen (BUN) level was assayed with the color metric QuantiChrom™ Urea Assay Kit (#DIUR-100, BioAssay Systems, Hayward, CA, USA).

### 4.5. Histology

Kidney tissue was placed into 10% buffered formalin, paraffin embedded, and sectioned at 3 µM before staining with Masson’s trichrome stain (MTS). MTS was performed by the University of Pittsburgh Medical Center Hillman Cancer Center and Tissue and Research Pathology/Pitt Biospecimen Core.

### 4.6. Immunoblot

Tissue homogenates were generated by douncing a portion of the recovered kidney in pre-chilled radioimmunoprecipitation buffer (RIPA: 50 mM Tris, pH 8.0; 150 mM NaCl; 0.1% SDS; 0.5% sodium deoxycholate; 1% Triton X-100) and 1x Halt^™^ Protease and Phosphatase Single-Use Inhibitor Cocktail (ThermoScientific, Rockford, IL, USA) and then centrifuged at 16,000× *g* for 15 min at 4 °C. Protein concentrations were determined with the Pierce™ BCA Protein Assay Kit (ThermoScientific). Samples were boiled in Laemmli sample buffer for 10 min. Equivalent protein concentrations were subjected to SDS-PAGE and transferred to PVDF membrane. The membrane was blocked in 5% nonfat milk for 1 h and then incubated overnight in primary antibody at 4 °C. After washes in TRIS-buffered saline with 0.1% Tween-20 (TBS-T) and incubation in horseradish peroxidase-conjugated secondary antibody for 1 h, detection of protein bands was performed with Pierce^™^ SuperSignal^®^ West Pico Chemiluminescent Substrate (ThermoScientific). Densitometry was performed with ImageJ (NIH, Bethesda, MD, USA).

### 4.7. Immunofluorescence

Tissue sections were deparaffinized with xylene and then hydrated through a graded series of ethanol. Antigen retrieval was performed with a heated citric acid-based solution (#H-3300, Vector Laboratories, Burlington, CA, USA). Blocking was performed for 1 h in 10% donkey serum before overnight incubation in primary antibodies (Nephrin and WT1) at 4 °C. The next day, the corresponding fluorescent-labeled secondary antibodies in 10% donkey serum were added for 2 h. Imaging was conducted using a Leica DM 6000B microscope or Leica TCS SP5 STED CW confocal microscope (Leica Microsystems Inc., Buffalo Grove, IL, USA).

### 4.8. Glomeruli Isolation

Mice were anesthetized and laparotomy–thoracotomy was performed. Intracardiac perfusion of Hank’s Balanced Salt Solution (HBSS) (#14025-076, Gibco Laboratories, Gaithersburg, MD, USA) was performed to exsanguinate the mice followed by perfusion with Dynabeads™ M-450 Tosylactivated (#14013, Invitrogen, Vilnius, Lithuania) diluted in Tris (0.2 M, pH 8.5; 0.1% BSA). Kidneys were harvested and then homogenized on ice. Homogenates were digested in HBSS with 10 mg/mL Collagenase type II (#17101-015, Gibco Laboratories) and DNase (#04716728001, Roche Diagnostics, Mannheim, Germany) for 1 h on an orbital shaker (37 °C, 160 rpm). Digested tissue was passed through a 100 µM cell strainer and washed with HBSS. Filtrate was centrifuged at 200× *g* for 5 min and the resulting pellet was resuspended in HBSS. Suspension was placed on a DynaMagnet (#12303D, Invitrogen, Oslo, Norway) and subjected to serial washes with HBSS to yield purified glomeruli.

### 4.9. Quantitative Real-Time PCR

Total RNA was extracted with TRIzol^®^ Reagent (Ambion^®^, Carlsbad, CA, USA) and subjected to reverse transcription with the RevertAid Reverse Transcriptase Kit (ThermoFisher Scientific, Pittsburgh, PA, USA). Reactions contained cDNA, iTAQ^™^ Universal SYBR^®^ Green Supermix (Bio-Rad Laboratories), nuclease-free water, and one of the following primer pairs: *Acta2* (actin alpha 2, smooth muscle) NM_007392.3 (Mouse) (Forward: 5′-GAGGCACCACTGAACCCTAA-3′; Reverse: 5′-CATCTCCAGAGTCCAGCACA-3′), *β-Actin* (actin beta) NM_007393.5 (Mouse) (Forward: 5′-ACACCCGCCACCAGTTC-3′; Reverse: 5′-TACAGCCCGGGGAGCAT-3′), *Col4a1* (collagen, type IV, alpha 1) NM_009931.2 (Mouse) (Forward: 5′-TCCGGGAGAGATTGGTTTCC-3′; Reverse: CTGGCCTATAAGCCCTGGT), *Cre* (Cre recombinase; enterobacteria phage P1) NC_005856.1 (Forward: 5′-AGCCGAAATTGCCAGGATCA-3′; Reverse: 5′-AACCAGCGTTTTCGTTCTGC-3′), *Fn1* (fibronectin) NM_010233.2 (Mouse) (Forward: 5′-CGAGGTGACAGAGACCACAA-3′; Reverse: 5′-CTGGAGTCAAGCCAGACACA-3′), *Mcp-3* (C-C motif chemokine ligand 7; monocyte chemoattractant protein-3) NM_013654.3 (Mouse) (Forward: 5′-AGGATCTCTGCCACGCTTC-3′; Reverse: 5′-TTGACATAGCAGCATGTGGAT-3′) [58], *Mcp-5* (C-C motif chemokine ligand 12; monocyte chemoattractant protein-5) NM_011331.3 (Mouse) (Forward: 5′-CCACCATCAGTCCTCAGGTATT-3′; Reverse: 5′-CGGACGTGAATCTTCTGCTT-3′) [58], *Nphs1* (nephrin) NM_019459.2 (Mouse) (Forward: 5′-CCCAGGTACACAGAGCACAA-3′; Reverse: 5′-CTCACGCTCACAACCTTCAG-3′), *Nphs2* (podocin) NM_130456.4 (Mouse) (Forward: 5′-CACTTTGGCCTGTCTTTGTG-3′; Reverse: 5′-GCCCAAGATGTAAAGGTTGC-3′). Quantitative PCR (qPCR) was performed using the CFX Connect^™^ Real-Time System (Bio-Rad Laboratories). Results were normalized to *β-Actin* and gene expression was determined with the comparative 2^−ΔΔCt^ method [59]. Melt curves were assessed to ensure specificity of a single product.

### 4.10. Statistical Analysis

To identify outliers, a ROUT outlier test was conducted. For survival analysis, a Kaplan–Meier curve was generated, and a log-rank test was used to compare survival distributions. For two independent group comparisons, an unpaired Student’s *t*-test (two-tailed) was performed. Each individual data point represents a mouse, and results were reported as mean ± S.E.M. GraphPad Prism 9.5.0 software (GraphPad Software Inc., La Jolla, CA, USA) was used. The threshold for significance was *p* < 0.05 with * *p* < 0.05, ** *p* < 0.01, *** *p* < 0.001, and **** *p* < 0.0001.

## 5. Conclusions

We did not find significant differences between control and knockout mice using two clinically relevant proteinuric models. Based on our findings, we propose that podocyte-specific MCP-1 production is not a major contributor to either Ang II- or Adr-induced proteinuric CKD as demonstrated by a lack of protection in the knockout mice. These results provide a valuable contribution to furthering the understanding of the role of podocyte-specific MCP-1 in CKD pathogenesis. Future studies will identify other sources of MCP-1 that play a role in disease pathogenesis.

## Data Availability

The analyzed data are contained within the manuscript.
